# The Cellular Prion Protein—ROCK Connection: Contribution to Neuronal Homeostasis and Neurodegenerative Diseases

**DOI:** 10.3389/fncel.2021.660683

**Published:** 2021-04-12

**Authors:** Benoit Schneider, Anne Baudry, Mathéa Pietri, Aurélie Alleaume-Butaux, Chloé Bizingre, Pierre Nioche, Odile Kellermann, Jean-Marie Launay

**Affiliations:** ^1^Inserm UMR-S1124, Paris, France; ^2^Université de Paris, Faculté des Sciences, Paris, France; ^3^Université de Paris - BioMedTech Facilities- INSERM US36 | CNRS UMS2009 - Structural and Molecular Analysis Platform, Paris, France; ^4^Inserm UMR 942, Hôpital Lariboisière, Paris, France; ^5^Pharma Research Department, Hoffmann-La-Roche Ltd., Basel, Switzerland

**Keywords:** prion, signaling, neuronal differentiation, cytoskeleton, inflammation, unfolded protein response, neurodegenerative diseases, amyloids

## Abstract

Amyloid-based neurodegenerative diseases such as prion, Alzheimer's, and Parkinson's diseases have distinct etiologies and clinical manifestations, but they share common pathological events. These diseases are caused by abnormally folded proteins (pathogenic prions PrP^Sc^ in prion diseases, β-amyloids/Aβ and Tau in Alzheimer's disease, α-synuclein in Parkinson's disease) that display β-sheet-enriched structures, propagate and accumulate in the nervous central system, and trigger neuronal death. In prion diseases, PrP^Sc^-induced corruption of the physiological functions exerted by normal cellular prion proteins (PrP^C^) present at the cell surface of neurons is at the root of neuronal death. For a decade, PrP^C^ emerges as a common cell surface receptor for other amyloids such as Aβ and α-synuclein, which relays, at least in part, their toxicity. In lipid-rafts of the plasma membrane, PrP^C^ exerts a signaling function and controls a set of effectors involved in neuronal homeostasis, among which are the RhoA-associated coiled-coil containing kinases (ROCKs). Here we review (i) how PrP^C^ controls ROCKs, (ii) how PrP^C^-ROCK coupling contributes to neuronal homeostasis, and (iii) how the deregulation of the PrP^C^-ROCK connection in amyloid-based neurodegenerative diseases triggers a loss of neuronal polarity, affects neurotransmitter-associated functions, contributes to the endoplasmic reticulum stress cascade, renders diseased neurons highly sensitive to neuroinflammation, and amplifies the production of neurotoxic amyloids.

## Introduction

The cellular prion protein (PrP^C^) garnered a lot of attention in the 90s because of its implication in a group of neurodegenerative diseases, Transmissible Spongiform Encephalopathies (TSEs), commonly named as prion diseases. TSEs affect both humans and animals. The most emblematic of these pathologies are Creutzfeldt-Jakob disease (CJD) and its variant (vCJD) in humans and Bovine Spongiform Encephalopathy (BSE), better known as mad cow disease (Houston and Andréoletti, 2019). TSEs are infectious diseases, and they can be transmitted between animals whether they belong to the same species or not. The crossing of the species barrier was highly publicized in the media with the transmission to humans (vCJD) of pathogenic prions (PrP^Sc^) present in the meat of cows at the root of the mad cow disease crisis at the end of the twentieth century (Lasmézas et al., 1996). These diseases are characterized by the spongiosis of the central nervous system, reactive gliosis, strong neuronal death, and the deposition in the brain of PrP^Sc^, possibly but not systematically forming plaques (Mikol, 1999; Soto and Satani, 2011).

PrP^Sc^ is the transconformational isoform of the normal PrP^C^. From a structural point of view, PrP^Sc^ is highly enriched in β-sheet secondary structures, while PrP^C^ is mainly composed of α-helices. The three-dimensional conversion of PrP^C^ into PrP^Sc^ causes prion diseases (Prusiner, 1982). PrP^C^ is endogenously expressed by all cells in the organism and is most abundantly present in neurons, notably at the synapse. PrP^C^ is a glycoprotein attached to the outer leaflet of the plasma membrane *via* a Glycosylphosphatidyl-Inositol (GPI) anchor and is oriented toward the extracellular space. The protein not only exerts ubiquitous functions but also displays some neurospecific roles as the toxicity of PrP^Sc^ is restricted to neurons and strictly depends on the expression of PrP^C^ in neurons. The conditional silencing of PrP^C^ specifically in neurons indeed protects mice from the deleterious effects of PrP^Sc^, despite the replication and accumulation of PrP^Sc^ in extraneuronal territories (Mallucci et al., 2003). For instance, two non-mutually exclusive models have been proposed to account for prion neurotoxicity: a PrP^C^ loss-of-function, for which PrP^C^ conversion into PrP^Sc^ cancels PrP^C^ functions in neurons, and a PrP^C^ gain-of-function, for which the interaction of PrP^Sc^ with PrP^C^ raises to toxic level signals physiologically coupled to PrP^C^ (Harris and True, 2006). These two scenarios would occur sequentially and/or concomitantly depending on the state of the pathology. Whatever the case, the identification of PrP^C^ roles in neurons is mandatory to grasp the molecular pathways of neurodegeneration at play in prion diseases and to identify potential therapeutic targets.

Unfortunately, mice knockout for PrP^C^ failed to reveal any obvious functions for PrP^C^ as they develop normally and do not exhibit major phenotypic abnormalities (Büeler et al., 1992; Manson et al., 1994; Castle and Gill, 2017). This is probably due to mechanisms occurring early during embryogenesis that compensate for the lack of PrP^C^ in mammals (Chadi et al., 2010). By contrast, experiments in zebrafish showed that the invalidation of PrP1 paralog provokes a gastrulation arrest and the invalidation of PrP2 paralog causes abnormal development of the nervous system (Málaga-Trillo et al., 2011), suggesting a critical role of PrP^C^ in embryogenesis and development. The minor phenotypic abnormalities of the different PrP KO mouse models, however, allowed proposing a role of PrP^C^ in the biology of stem cells, a cytoprotective effect of PrP^C^ against several stresses, or a role of PrP^C^ in the interaction between the axon of neurons and Schwann cells in peripheral nerves (Chiarini et al., 2002; Weise et al., 2006; Zhang et al., 2006; Bremer et al., 2010; Gourdain et al., 2012; Bravard et al., 2015). Several cell paradigms were also very helpful in unraveling PrP^C^ functions. With the help of diverse neuronal cell lines (N2a neuroblastoma cells, PC12 pheochromocytoma cells, 1C11 neuronal stem cells and their serotonergic or noradrenergic neuronal progenies, SH-SY5Y dopaminergic neurons…) and primary cultures of neurons (cerebellar granule neurons, cortical neurons, hippocampal neurons), a multifaceted function was proposed for PrP^C^, dealing with cell adhesion, neuritogenesis and stability of neurite extensions, neuronal differentiation, regulation of cell redox equilibrium, stress protection, neuronal survival, regulation of neurotransmitter-associated functions, and neuronal plasticity. Such a diversity of PrP^C^ functions relates to the general implication of PrP^C^ in multiple cell signaling pathways (Mouillet-Richard et al., 2000a; Schneider et al., 2011). In lipid-rafts of the plasma membrane, PrP^C^ plays the role of a cell surface receptor or co-receptor, or a scaffolding protein that controls the dynamic assembly of signaling modules, downstream regulating signaling pathways, and cell biological functions (Linden et al., 2008). Among the effectors governed by PrP^C^ are the RhoA-associated coiled-coil containing kinases (ROCKs), a group of two Serine/Threonine kinases named ROCK-1 (Rho-kinase β/ROKβ) and ROCK-2 (Rho-kinase α/ROKα) (Nakagawa et al., 1996). ROCK-1 and ROCK-2 human genes are located on chromosome 18 (18q11.1) and chromosome 2 (2p24), respectively. Mammalian ROCK-1 (1,354 amino acids) and ROCK-2 (1388 amino acids) exhibit 64% overall identity in amino acid sequences, with 92% identity in the kinase domain. The two ROCK isoforms are ubiquitously expressed with different relative levels depending on the tissue and cell type. Both ROCK-1 and ROCK-2 are expressed in the brain and found in neurons with, however, specifical subcellular distribution depending on the ROCK isoform (González-Forero et al., 2012). Physiologically, ROCKs have been implicated in the regulation of neuronal polarity, survival, and functions. Interestingly, an upregulation of ROCK expression in the brain is associated with aging and amyloid-based neurodegenerative diseases (Koch et al., 2018).

Here, we review the molecular mechanisms by which PrP^C^ signaling controls ROCKs and the implication of such coupling in neuronal differentiation and homeostasis. We will also discuss the pathological implication of the PrP^C^-ROCK pathway and downstream effectors in prion diseases, but also in other unrelated neurodegenerative diseases, such as Alzheimer's and Parkinson's diseases, for which PrP^C^ was reported to relay, at least in part, the neurotoxicity of β-amyloids (Aβ) and pathological α-synuclein, respectively.

## PrP^C^ and Rock: A Functional Partnership for Neuronal Differentiation

Neuronal differentiation is a complex phenomenon occurring during development and in adulthood that permits for a neuronal stem cell to acquire a polarized morphology with the cell body, dendrites and an axon, and to implement neurotransmitter-associated functions, that is, the capacity to synthesize, store, degrade, and transport the neurotransmitter. Beyond the extrinsic and intrinsic cues, the genetic programs, and the repertoire of transcription factors involved in the commitment and differentiation of neuronal stem cells into neurons, deep modifications of the cytoskeleton in neuronal stem cells are mandatory for neuronal polarization and functions. This includes a severe reorganization and activation of focal adhesions combined to focalized instability of the actin cytoskeleton in the stem cell sphere to ensure the initial sprouting of neurites, that is, the formation of a bud with the protrusion of microtubules. The bud then evolves in a neurite, before specialization in an axon or a dendrite (da Silva and Dotti, 2002; Cingolani and Goda, 2008; Witte and Bradke, 2008).

**Figure 1:**
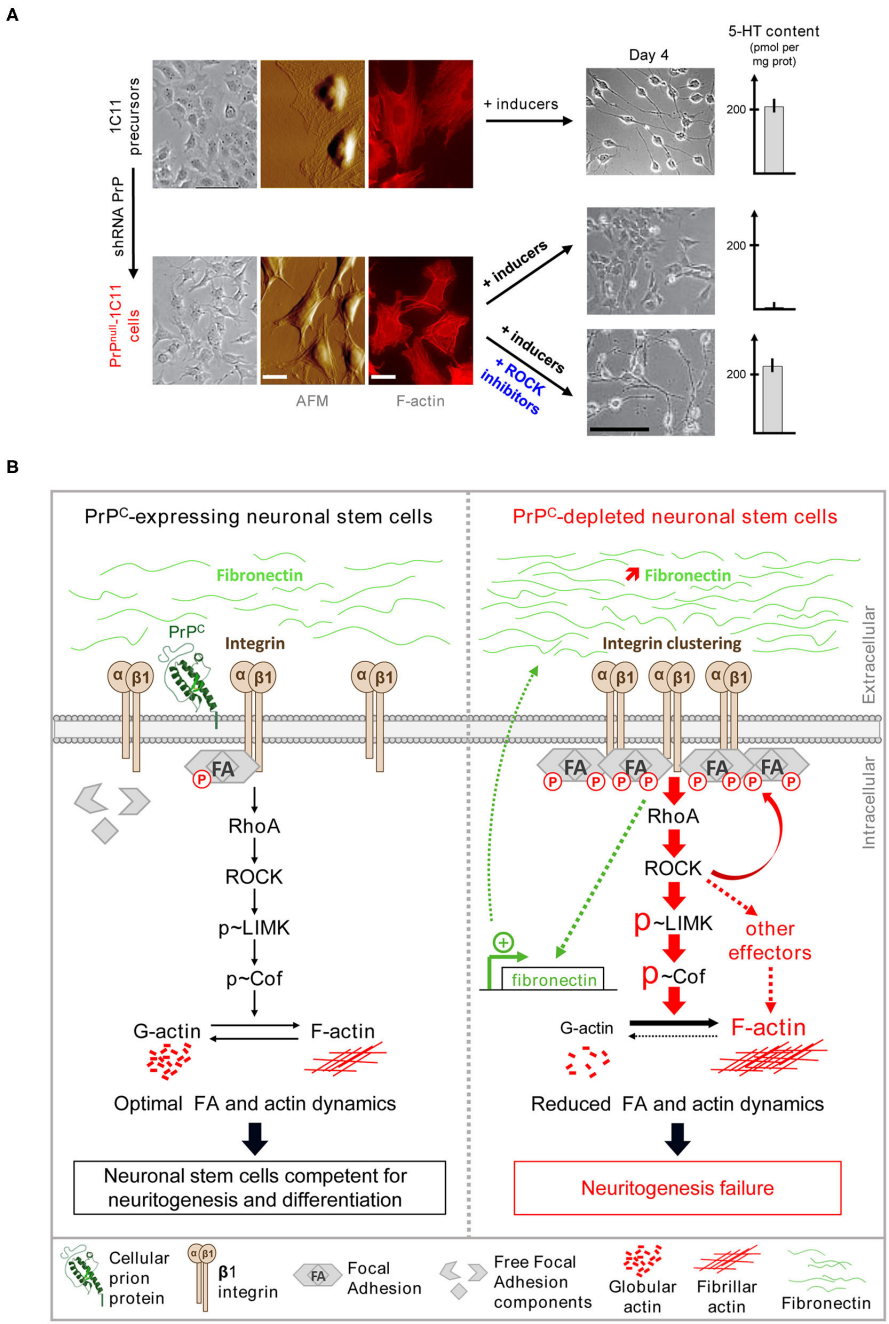
PrP^C^ renders neuronal stem cells competent for neuronal differentiation by toning down the β1 integrins/ROCK signaling. **(A)** Stable PrP^C^ depletion in 1C11 neuronal stem cells impairs neuritogenesis and the implementation of neuronal functions in a ROCK-dependent manner. shRNA-mediated chronic PrP^C^ depletion in 1C11 cells (referred to as PrP^null^-1C11 cells) alters cell morphology as revealed by phase contrast microscopy and Atomic force microscopy (AFM) deflection images. Actin staining shows an increase of actin stress fibers in PrP^null^-1C11 cells. Upon exposure to serotonergic inducers, PrP^null^-1C11 cells fail to implement neurites and synthesize serotonin. Pharmacological ROCK inhibition with Y-27632 or dimethylfasudil restores neuritogenesis and acquisition of neuronal functions in PrP^null^-1C11 cells exposed to serotonergic inducers. **(B)** By limiting plasma membrane integrin microclustering and downstream RhoA/ROCK/LIMK/cofilin signaling, PrP^C^ ensures optimal focal adhesion and actin dynamics. This negative regulatory role of PrP^C^ toward ROCK is necessary for neurite sprouting and the onset of neuronal functions **(left)**. Depletion of PrP^C^ provokes β1 integrin clustering and downstream overactivation of RhoA/ROCK/LIMK/cofilin signaling. Overphosphorylated cofilin loses its severing activity toward fibrillar actin at the root of neuritogenesis failure in PrP^null^-1C11 cells. β1 integrin clustering and excessive signaling is self-sustained by the overproduction of the β1 integrin ligand, fibronectin **(right)**.

PrP^C^ was shown to influence neurite outgrowth. Neurons isolated from the brain of PrP KO mice exhibit shorter neurites than those expressing PrP^C^ (Kanaani et al., 2005). Furthermore, on exposure to recombinant PrP^C^, embryonic hippocampal neurons rapidly polarize and develop synapses. At the plasma membrane, the interaction of PrP^C^ with neural cell adhesion molecules (NCAMs) potentiates neuritogenesis in hippocampal neurons, and the interaction of PrP^C^ with proteins of the extracellular matrix (ECM), such as laminin and vitronectin, sustains neurite outgrowth (Santuccione et al., 2005; Coitinho et al., 2006; Hajj et al., 2007). The sprouting of neurites was also shown to depend on PrP^C^ and control of β1 integrins coupling to ROCK effectors (Loubet et al., 2012; Alleaume-Butaux et al., 2013). This latter study relies on the characterization of PrP^C^ roles in the 1C11 neuronal stem cell line. 1C11 precursor cells display a stable neuroepithelial phenotype and do not differentiate spontaneously even in the presence of 10% fetal calf serum (Buc-Caron et al., 1990). Upon appropriate induction, almost 100% of 1C11 neuronal stem cells engage in either a serotonergic (1C11^5−HT^) or a noradrenergic (1C11^NE^) program (Mouillet-Richard et al., 2000b). Differentiating 1C11 neuronal stem cells develop neurites and acquire all the neurotransmitter-associated functions with precise kinetics in a synchronous manner. Whatever the cell differentiation state, PrP^C^ is endogenously expressed at similar levels. On stable PrP^C^ silencing in 1C11 precursor cells, PrP^null^-1C11 cells fail to implement neurites and neuronal functions upon exposure to serotonergic or noradrenergic inducers and keep an epithelial-like phenotype (Figure 1A). The defect in neurite sprouting in PrP^null^-1C11 cells originates from a loss of the PrP^C^ regulatory role toward β1 integrins at the plasma membrane (Loubet et al., 2012). The silencing of PrP^C^ provokes cell surface micro-clustering of β1 integrins and a rise of β1 integrin signaling. Such a modification of β1 integrin activity leads to the excessive recruitment and the overstimulation of ROCKs by small G-protein RhoA (for Ras homolog gene family member A). Overactivated ROCKs then promote the phosphorylation of LIM kinases (LIMKs) 1 and 2 at Thr505 and Thr508, respectively, raising the enzymatic activity of LIMKs (Ohashi et al., 2000; Sumi et al., 2001). Overactivated LIMKs, in turn, catalyze the phosphorylation of cofilin at Ser3, which impairs the severing activity of cofilin toward fibrillar actin (F-actin) (Arber et al., 1998). The deregulation of the β1 integrin-RhoA-ROCK-LIMK-cofilin pathway in PrP^null^-1C11 cells alters the actin cytoskeleton architecture with tensed F-actin cables and with reduced turnover compared to PrP^C^ expressing 1C11 cells (Figure 1B) (Loubet et al., 2012). Kim et al. confirmed such a regulatory role of PrP^C^ over RhoA, ROCK, and LIMK activities in PC12 cells induced to differentiate into neurons, hippocampal neuronal cells, as well as in the brain of transgenic mice expressing variable levels of PrP^C^ (Kim et al., 2017). Apart from β1 integrins, the control of RhoA activity by PrP^C^ would also depend on an interaction between PrP^C^ and RhoA and a possible enhanced PrP^C^-mediated interaction between RhoA and p190RhoGAP, a GTPase-activating protein, which inactivates RhoA (Kim et al., 2017).

Moreover, several reports incriminate the RhoA-ROCK-LIMK-cofilin pathway in the rise of phosphorylation and clustering of miscellaneous components of Focal Adhesions (FAs), including Src, FAK, and paxillin (Chrzanowska-Wodnicka and Burridge, 1996; Murakami et al., 1999). Loss of the PrP^C^ modulatory role on the β1 integrin-RhoA-ROCK-LIMK-cofilin cascade would additionally account for the excessive stability of FAs in PrP^null^-1C11 cells (Loubet et al., 2012). In this context, the ROCK-dependent stiffening of the actin cytoskeleton and the reduced renewal of FAs both contribute to the neuritogenesis impairment caused by the absence of PrP^C^ in neuronal stem cells. Because many other cytoskeleton-associated proteins, including tau, MAP2, neurofilament, the ERM proteins ezrin, radixin, and moesin…, were reported as ROCK substrates (Koch et al., 2018), the fact that the ROCK-dependent phosphorylation of those protagonists in the absence of PrP^C^ also contributes to neuritogenesis defect should not be excluded.

Furthermore, the pharmacological inhibition of ROCKs with either Y-27632 or dimethylfasudil is sufficient to restore the sprouting of neurites by PrP^null^-1C11 cells (Figure 1A) or PrP^null^-PC12 cells when exposed to neuronal inducers (Loubet et al., 2012), by relaxing the actin cytoskeleton and rescuing the turnover of FAs. The sprouting of neurites, and therefore neurite outgrowth, that normally occurs during development and in an adult, thus relies on the ability of PrP^C^ to limit the activity of ROCKs by organizing spatially β1 integrins at the plasma membrane and inactivating small G-protein RhoA. Through control of the intensity of the β1 integrins-RhoA-ROCK signaling pathway, PrP^C^ thus renders neuronal stem cells competent for differentiation and neuritogenesis (Figure 1B) (Alleaume-Butaux et al., 2013).

Beyond neurite sprouting and outgrowth, the regulation of ROCK activity by PrP^C^ also governs the implementation of neuronal functions that accompanies neuronal differentiation. In polarized neurons, the expression of neuronal functions relies on the trafficking of specific mRNAs and proteins to subcellular territories at a distance from the soma, an event driven by the actin cytoskeleton (Mooseker et al., 1984; Jung et al., 2012). Moreover, it has been shown that the actin cytoskeleton additionally plays the role of a physical organizer and regulator of mRNAs translation by ribosomes (Heuijerjans et al., 1989; Kim and Coulombe, 2010). Of note, mRNAs encoding serotonergic and noradrenergic-associated functions are present in 1C11 stem cells, but they are dormant. The absence of PrP^C^ does not affect neuronal mRNAs as they are expressed in PrP^null^-1C11 cells to a level highly comparable to that measured in PrP^C^-expressing 1C11 cells. Relaxing the actin cytoskeleton and unlocking neuritogenesis in PrP^null^-1C11 cells on ROCK inhibition with dimethylfasudil or Y-27632 allow for the translation of mRNAs encoding neurotransmitter-associated functions upon exposure of PrP^null^-1C11 cells to neuronal inducers (Figure 1A), suggesting rescued trafficking of neuronal mRNAs in growing neurites. The contribution of the PrP^C^-ROCK cascade to the expression of neuronal functions would also relate to a modulatory action of ROCKs on initiation factors involved in mRNA translation (see below).

## PrP^C^ and Rock: A Duo of Neuroprotection Against TNFα Inflammation

Another effector downstream of ROCKs drastically impacted by the absence of PrP^C^ is 3-phosphoinositide-dependent kinase 1 (PDK1), an enzyme critically involved in the neuronal cell response to the pro-inflammatory cytokine TNFα (Ezpeleta et al., 2017). While primarily known for its role in the immune system, TNFα physiologically regulates the intensity of neuronal functions (synthesis/degradation of the neurotransmitter) in the nervous system and the firing activity of autoreceptors (Ignatowski et al., 1997; Hayley et al., 1999; Pietri et al., 2005; Pradines et al., 2009). TNFα has also been associated with memory processes with the modulation of the long-term potentiation and its consolidation (Golan et al., 2004). The non-toxic action of this pro-inflammatory cytokine toward neurons depends on strict control of the production level of TNFα by neurons (autocrine) or microglia (paracrine) and the competence (intrinsic sensitivity) of neuronal cells to respond to TNFα.

**Figure 2:**
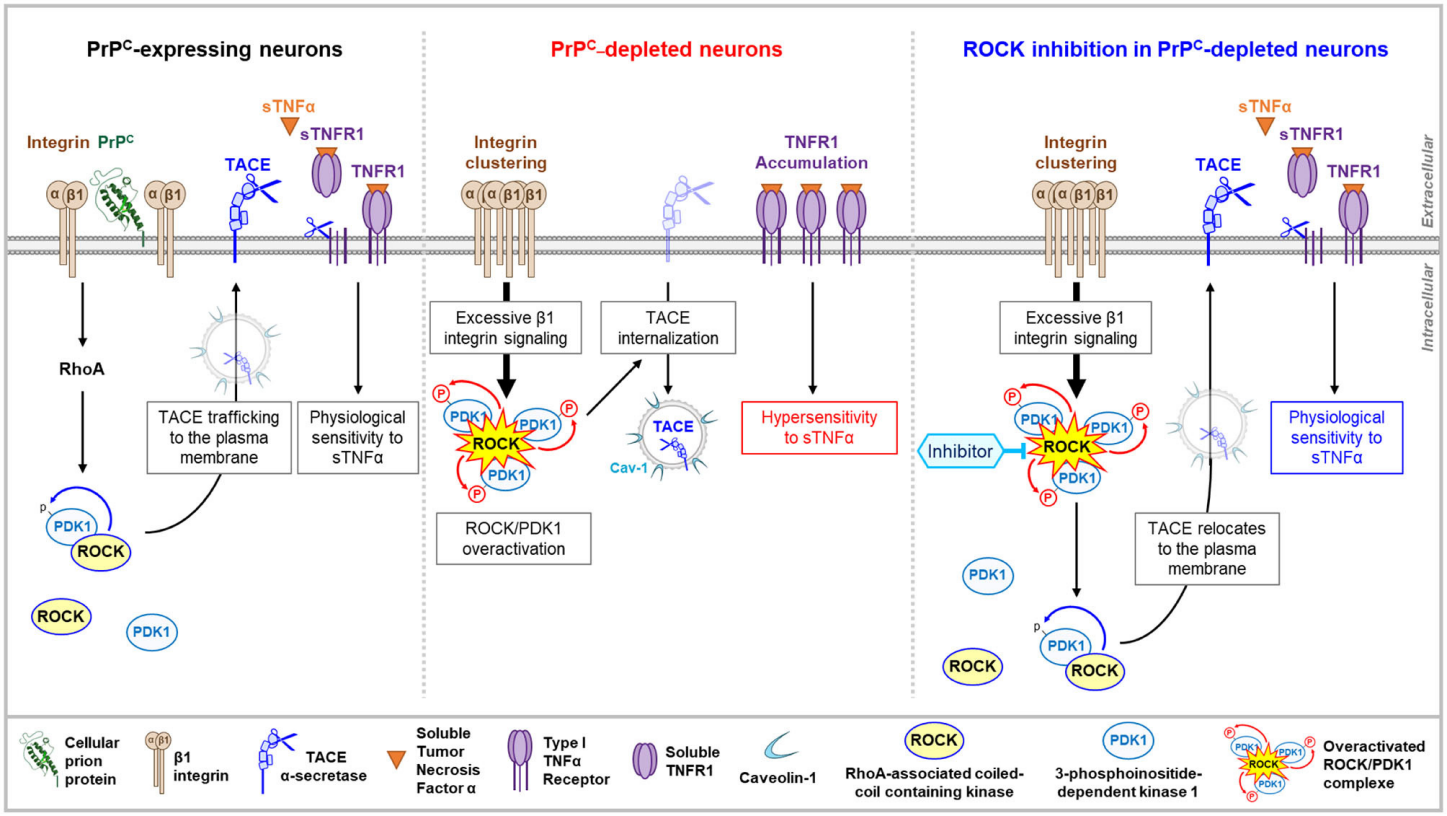
PrP^C^–ROCK connection controls TACE trafficking and cell sensitivity to TNFα. By toning down the integrin-dependent RhoA activation, PrP^C^ lowers the enzymatic activity of the ROCK/PDK1 complex that permits the α-secretase TACE to stay at the plasma membrane. TACE cleaves cell surface TNFR1 and releases sTNFR1 that neutralizes sTNFα and thereby limits the cell sensitivity to sTNFα **(left)**. In PrP^C^-depleted neurons, the overactivation of ROCK/PDK1 module provoked by the micro-clustering of β1 integrins triggers TACE internalization and neutralization in caveolin-1 enriched vesicles. Internalized TACE is uncoupled from TNFR1 substrate that accumulates at the cell surface and renders PrP^C^-depleted neurons hypervulnerable to sTNFα toxicity **(middle)**. In PrP^C^-depleted neurons, pharmacological inhibition of ROCK decreases PDK1 activity, which permits TACE to locate back to the plasma membrane and to restore TACE shedding activity toward TNFR1 **(right)**.

PrP^null^-1C11 cells, 1C11^5−HT^ serotonergic neurons silenced for PrP^C^ expression, as well as primary cultures of cerebellar granule neurons isolated from PrP KO mice exhibit higher sensitivity to TNFα than their normal counterparts expressing PrP^C^ (Ezpeleta et al., 2017). Such an increase in TNFα cell sensitivity originates from the accumulation of TNFα death receptor type 1 (TNFR1) at the plasma membrane of PrP^null^-cells and a reduced amount of soluble TNFR1 (sTNFR1) that neutralizes TNFα in the surrounding milieu of those cells (Figure 2). The imbalance in cell surface TNFR1 over the sTNFR1 ratio is associated with a deficit of TNFR1 shedding by the α-secretase TACE (aka ADAM17). While most present at the plasma membrane of PrP^C^ expressing neurons, TACE is internalized in PrP^C^-depleted cells and accumulates in microvesicles enriched with the caveolin-1 protein (Figure 2). TACE is then neutralized, as it is no longer available at the plasma membrane to catalyze the cleavage of TNFR1 into sTNFR1, accounting for the augmented sensitivity of PrP^C^-depleted cells to TNFα. The kinase PDK1, whose enzymatic activity is enhanced in the absence of PrP^C^, was shown to trigger the internalization of TACE (Ezpeleta et al., 2017). The increase in PDK1 activity in PrP^null^-cells depends upstream on the rise of β1 integrin-ROCK signaling (Figure 2). Pharmacological inhibition of ROCKs in PrP^null^-cells reduces PDK1 activity that returns to the basal level measured in PrP^C^-expressing cells. This authorizes TACE to target back the plasma membrane of PrP^null^-cells where it recovers its shedding activity toward TNFR1, restoring a physiological sensitivity to TNFα (Figure 2) (Ezpeleta et al., 2017). PrP^C^ thus exerts cytoprotection against TNFα inflammatory stress in neurons by forcing the α-secretase TACE to stay at the plasma membrane. This renders TACE competent for TNFR1 cleavage, sTNFR1 release, and neutralization of the TNFα pro-inflammatory cytokine. As for neurite sprouting, the protective role of PrP^C^ against TNFα depends on the ability of PrP^C^ to limit the micro-clustering of β1 integrins, to tone down the intensity of β1 integrins coupling to ROCKs, and subsequently, the ROCK-dependent activation of PDK1 (Figure 2).

At a molecular level, how do ROCKs regulate PDK1 activity? According to the literature, the augmentation in PDK1 activity depends on several steps: (i) translocation and docking of cytosolic PDK1 to the PIP_3_ phospholipid at the inner leaflet of the plasma membrane, (ii) phosphorylations, (iii) conformational changes, and (iv) binding of effectors such as HSP90 or Src kinases for additional phosphorylations (Li et al., 2010; Calleja et al., 2014). In this complex regulatory network, ROCK-1 contributes to the rise in PDK1 activity by forming a molecular complex with PDK1 (Pinner and Sahai, 2008; Alleaume-Butaux et al., 2015; Ezpeleta et al., 2017) and phosphorylating PDK1. Phosphorylation of PDK1 by ROCK-1 improves the stability of the ROCK-PDK1 complex. Supporting this, the inhibition of ROCKs with Y-27632 or dimethylfasudil prevents the phosphorylation of PDK1, which is accompanied by a reduction in the amount of the ROCK-PDK1 complex in neuronal cells. Of note, phosphorylation of PDK1 by ROCK-1 fits within a phosphorylation sequence, in which ROCK-mediated phosphorylation of PDK1 follows PDK1 autophosphorylation at Ser241. PDK1 Ser241Ala mutation indeed avoids PDK1 autophosphorylation, which thus impairs the formation of the ROCK-PDK1 complex, the ROCK-mediated phosphorylation of PDK1, and thereby, the rise in PDK1 activity.

The ROCK-dependent increase in PDK1 activity would also rely on the capacity of ROCK to favor the docking of PDK1 to PIP_3_ phospholipid at the inner face of the plasma membrane. PIP_3_ originates from the phosphorylation of PI(4,5)P_2_ by PI3kinase (PI3K). The production of PIP_3_ however, depends on the unmasking of PI(4,5)P_2_ that renders the phospholipid available for PI3K activity. PI(4,5)P_2_ is naturally protected by a family of proteins, the myristoylated alanine-rich C kinase substrates (MARCKS) that attach to the inner leaflet of the plasma membrane through electrostatic bonds, and aromatic amino-acids, and myristoyl groups that insert into the plasma membrane (Brudvig and Weimer, 2015). Of note, MARCKS is a direct substrate of ROCKs (Ikenoya et al., 2002). The phosphorylation of MARCKS by ROCKs introduce negative charges on MARCKS that then loses affinity for plasma membrane phospholipids and provokes MARCKS removal from the plasma membrane. This renders PI(4,5)P_2_ accessible for PI3K and conversion into PIP_3_, thus possibly contributing to the rise in PDK1 activity. Such a mechanism would contribute to the increase in PDK1 activity in PrP^null^-cells, in which the absence of PrP^C^ has been shown to negatively impact on the MARCKS protein level (Mehrabian et al., 2016) in addition to overstimulating ROCK activity *via* the β1 integrins (Ezpeleta et al., 2017).

These whole data illustrate that PrP^C^ fine-tunes the neuronal cell sensitivity to the pro-inflammatory cytokine TNFα through a negative control of the β1 integrin-ROCK-PDK1 signaling pathway. This ensures the correct trafficking and localization of the α-secretase TACE at the plasma membrane, where it is fully available for catalyzing the cleavage of transmembrane TNFRs. As TACE admits many other substrates, including pro-TNFα, p75 neurotrophic receptor, PrP^C^ itself (Vincent et al., 2001; Edwards et al., 2008; Gooz, 2010), the PrP^C^-ROCK/PDK1 signaling pathway will adjust the production of neurotrophic factors and regulators of the neuronal activity and manage the neuronal cell response to those signals, overall contributing to the global homeostasis of neurons.

## Prion Infection, Rock Oversignaling, and Polarity Alterations

**Figure 3:**
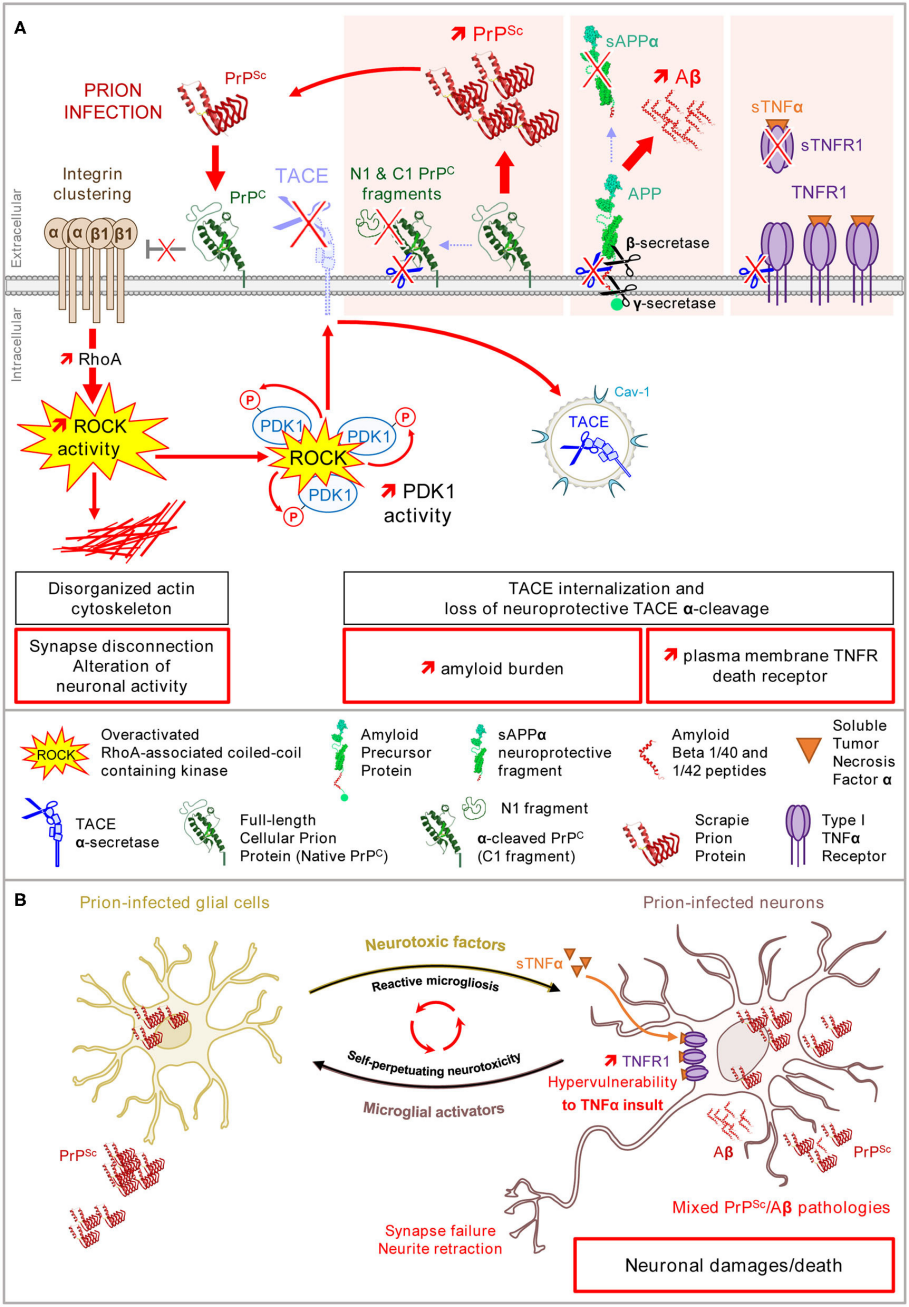
Loss of PrP^C^ control of β1 integrin–ROCK signaling upon prion infection alters neuronal polarity, renders neurons highly sensitive to TNFα, and promotes PrP^Sc^ and Aβ accumulation. **(A)** In prion-infected neurons, PrP^Sc^ provokes a loss of PrP^C^ negative regulatory role on β1 integrin/ROCK signaling. Overactivated ROCKs (i) affect the neuronal polarity and connectivity *via* the LIMK/cofilin/actin pathway, (ii) favor plasma membrane TNFR accumulation, and (iii) amplify the production of PrP^Sc^ and Aβ *via* PDK1 overactivation and TACE internalization. **(B)** In the brain, prion-infected glial cells release neurotoxic factors such as TNFα that would precipitate the death of prion-infected neurons primed by the ROCK-dependent accumulation of TNFR1 at the plasma membrane. The ROCK-dependent rise of PrP^Sc^ and Aβ peptides would self-sustain the toxic dialog between microglial cells and neurons (microglial reaction).

**Figure 4:**
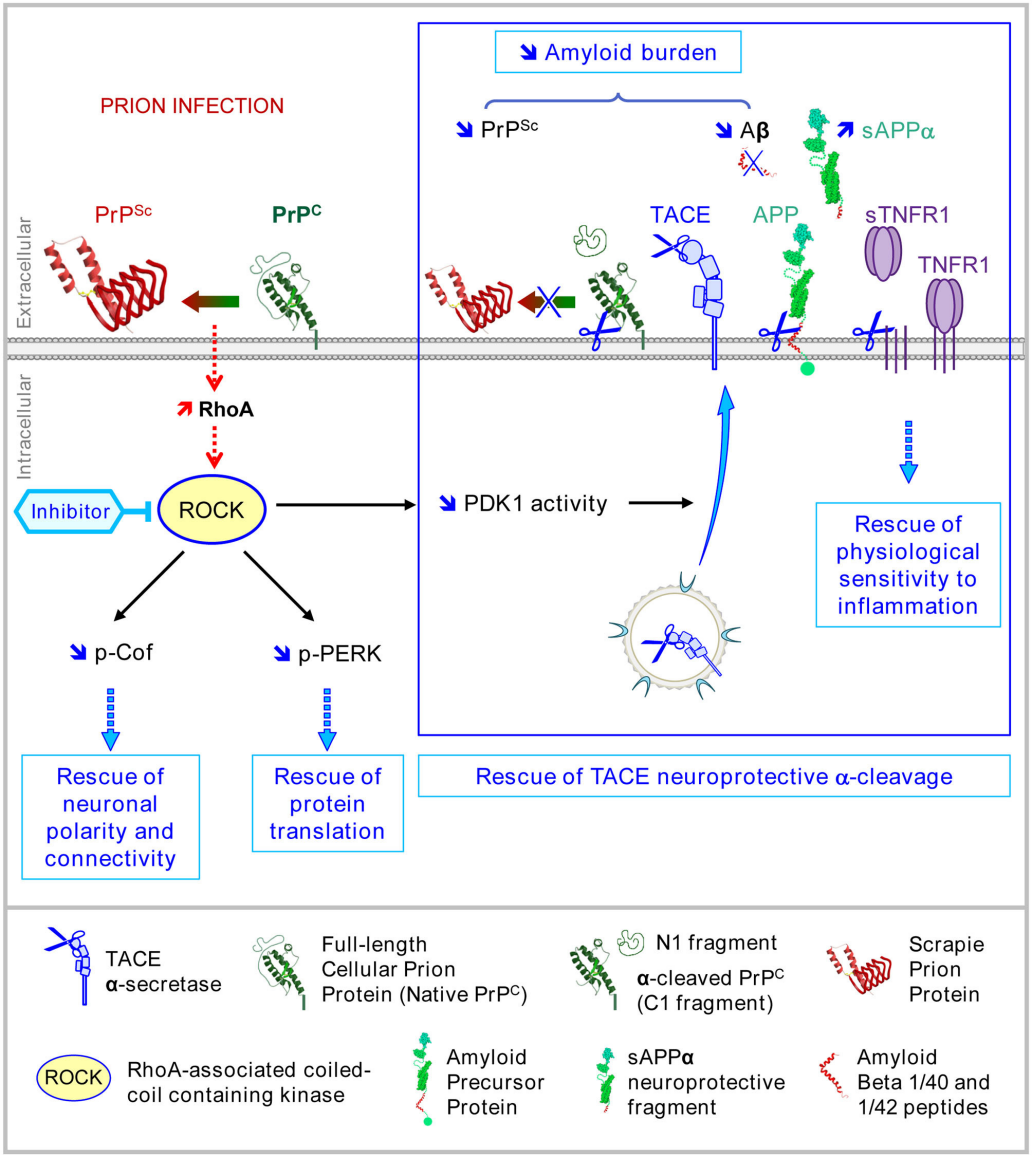
Multiple beneficial effects afforded by ROCK inhibition in prion diseases. In prion diseases, the pharmacological inhibition of ROCKs with Y-27632 or dimethylfasudil counteracts PrP^Sc^ neurotoxicity by rescuing (i) the neuronal polarity and connectivity through the restoration of cofilin-mediated actin severing, (ii) eIF2α-dependent protein translation through attenuation of PERK signaling, and (iii) the neuroprotective TACE α-secretase activity toward three main substrates: TNFR1, which restores physiological sensitivity to TNFα, PrP^C^, and APP, which limits the accumulation of neurotoxic amyloids, i.e., PrP^Sc^ and Aβ.

As outlined above, the neurotoxicity of pathogenic prions PrP^Sc^ depends on the interaction of PrP^Sc^ with PrP^C^ and the corruption of PrP^C^ signaling that generates toxic signals for neurons. This concerns the PrP^C^-mediated control of the β1 integrin-ROCK pathway (Figure 3A) (Alleaume-Butaux et al., 2015). 1C11 precursor cells, as well as cerebellar granule neurons grown in culture, and infected with prions, display clustered β1 integrins at the plasma membrane, dense focal adhesions with slow turnover, and rigid F-actin fibers. Such cytoskeleton abnormalities impact on neuronal differentiation of prion-infected 1C11 stem cells; those remain flat with short neurite extensions. This also provokes synapse failure and axon retraction in a reconstructed network of cortical and striatal mature neurons (Deleglise et al., 2013) infected with prions (Alleaume-Butaux et al., 2015). The inhibition of ROCKs restores neuritogenesis in prion-infected 1C11 stem cells and preserves the network of cortical and striatal neurons infected with prions from synapse disconnection and axon retraction (Figure 4). From a molecular point of view, the overactivation of ROCKs in neuronal stem cells and mature neurons infected with prions, affect the neuronal polarity and connectivity by altering the dynamics of both FAs and the actin cytoskeleton, the latter being necessary to sheath the axon and to stabilize the synaptic button (Figures 3A,B). Beyond *in vitro* neuronal cell models, ROCKs oversignaling was also evidenced *in vivo* in the brain of prion-infected mice according to cofilin overphosphorylation (Alleaume-Butaux et al., 2015; Kim et al., 2020) and was confirmed by a kinomics approach (Shott et al., 2014). In 2018, Zafar et al. provided evidence for altered cofilin and deregulation of upstream regulators (ROCK-2, LIMK1) in the cortex and cerebellum of humans with sporadic Creutzfeldt-Jakob disease (Zafar et al., 2018).

At a functional level, prion-infected 1C11 cells differentiated into serotonergic or noradrenergic neuronal cells with the help of the ROCK inhibitors express neuronal functions to a level comparable to that measured in pre-infectious conditions, indicating that ROCK inhibitors exert protection against the detrimental effect of PrP^Sc^ on neurotransmitter-associated functions (Alleaume-Butaux et al., 2015). Again, this may relate to the rescued coordination between neuronal morphogenesis and the translation of mRNAs encoding neuronal functions. Importantly, there is a strong reduction of oxidized derivatives of monoamines, i.e., neurotoxins (Mouillet-Richard et al., 2008), in prion-infected serotonergic 1C11^5−HT^ and noradrenergic 1C11^NE^ neuronal cells differentiated in the presence of Y-27632 or dimethylfasudil (Alleaume-Butaux et al., 2015), suggesting that the inhibition of ROCK exerts an anti-oxidant activity (Guo et al., 2013; Gibson et al., 2014). How ROCKs and ROCK inhibitors influence the redox equilibrium in neurons remains elusive. Interestingly, PrP^Sc^ has been shown to overactivate NADPH oxidase, a generator of reactive oxygen species (ROS) (Schneider et al., 2003; Pietri et al., 2006). The excessive NADPH oxidase-dependent production of ROS combined with a depletion of antioxidant systems contributes to the onset of oxidative stress conditions that threaten the integrity and homeostasis of prion-infected neurons. Notably, this (i) poisons redox-sensitive enzymes involved in the synthesis of monoamines (serotonin, norepinephrine, dopamine), (ii) is at the root of the production of the oxidized derivatives of monoamines, (iii) activates stress kinases such as p38 and JNK MAP kinases (Pradines et al., 2013), and (iv) affects the interaction between neurons and the extracellular matrix, i.e., laminin (Ermonval et al., 2009) and fibronectin (Loubet et al., 2012), thus placing prion-infected neurons on the path to degenerate. As functional links between ROCK and NADPH oxidase were evidenced in immune cells (Filina et al., 2014) and the heart (Zhang et al., 2018), the beneficial effect of ROCK inhibitors toward oxidative stress in prion-infected neurons would relate to the down-regulation of NADPH oxidase activity.

## Prion Infection, Rock Oversignaling, and Neuronal Hypersensitivity to Inflammation

PrP^Sc^-induced corruption of the PrP^C^ negative regulatory function toward β1 integrin signaling also causes ROCK-mediated PDK1 overstimulation (Figure 3A). The overactivation of ROCK-1 induced by PrP^Sc^ increases the amount of PDK1 engaged in the ROCK-PDK1 complexes and phosphorylated by ROCK-1, leading to an increase in PDK1 activity, and thereby the internalization of TACE that loses its protective shedding activity toward plasma membrane TNFR1. The ROCK-dependent accumulation of TNFR1 at the plasma membrane of prion-infected neurons does not promote neuron death. It constitutes a priming event that would precipitate the death of prion-infected neurons following brain inflammation (Figure 3B). It is worth noting that neuroinflammation in prion diseases is atypical-qualified (Perry et al., 2002), as the level of pro-inflammatory factors, including TNFα, released by prion-infected microglial cells (astrocytes) along with the microglial reaction is rather low compared to other amyloid-based neurodegenerative diseases. Even if the level of TNFα present in the microenvironment of prion-infected neurons remains low, the ROCK-induced rise in the intrinsic vulnerability of prion-infected neurons to TNFα contributes to neurodegeneration. By rescuing the TACE shedding activity toward TNFR at the plasma membrane, the inhibition of ROCKs desensitizes prion-infected neurons from TNFα insult (Figure 4) and thus counteracts prion neurotoxicity (Alleaume-Butaux et al., 2015).

## Prion Infection, Rock Oversignaling, and Accumulation of Neurotoxic PrP^Sc^ and Aβ Peptides: Mixed PrP^Sc^/Aβ Pathologies in Prion Diseases

A second detrimental effect of the overactivation of ROCK and PDK1 within a prion-infectious context is the active replication and accumulation of pathogenic prions PrP^Sc^ in neurons. The replication of PrP^Sc^ is a complex phenomenon in which PrP^C^ acquires the three-dimensional structure of PrP^Sc^ upon the interaction of PrP^C^ with PrP^Sc^ (Pan et al., 1993). Of note, several PrP^C^ isoforms co-exist at the cell surface of healthy neurons with full-length PrP^C^ (also called native PrP^C^) and truncated PrP^C^ (also called C1 fragment), each of these PrP^C^ isoforms being non-, mono-, or bi-glycosylated (Chen et al., 1995). Truncated PrP^C^ originates from the α-cleavage of full-length PrP^C^ between residues 111 and 112 by α-secretases, such as TACE (Vincent et al., 2001; Taylor et al., 2009). Several studies report that full-length PrP^C^ is highly prone to conversion into PrP^Sc^, while truncated PrP^C^ resists to transconformational changes and exerts a dominant negative effect on the conversion of full-length PrP^C^ into PrP^Sc^ (Westergard et al., 2011; Westergard and True, 2014). In prion-infected neurons, TACE internalized in response to the overactivation of ROCK-PDK1 kinases also loses its protective α-cleavage activity toward full-length PrP^C^ (Pietri et al., 2013; Alleaume-Butaux et al., 2015). This maintains PrP^C^ in its full-length form, thus amplifying PrP^C^ conversion into PrP^Sc^ and thereby the accumulation of neurotoxic PrP^Sc^ (Figure 3A).

A third consequence of TACE internalization downstream of overactivated ROCK/PDK1 in prion-infected neurons is the uncoupling of TACE activity to the amyloid precursor protein (APP) (Ezpeleta et al., 2019). Internalized TACE is no longer available at the plasma membrane to execute the non-amyloidogenic α-cleavage of APP into the neuroprotective fragment sAPPα (Figure 3A). This instead favors the amyloidogenic cleavage of APP into Aβ40/42 by β- and γ-secretases. Beyond PrP^Sc^ replication, the deregulation of the ROCK/PDK1-TACE pathway thus promotes the accumulation of a second type of neurotoxic amyloid proteins, the Aβ peptides (Figure 3A). The ESI-IM-MS characterization of those Aβ peptides generated by prion-infected neurons reveals that Aβ40/42 peptides are produced mainly as monomers, but trimers and tetramers of Aβ40/42 are also generated (Ezpeleta et al., 2019). Of note, trimers of Aβ40/42 are Aβ entities that are highly aggregative and neurotoxic (Lesné et al., 2006; Condello and Stöehr, 2018). Co-inoculated with PrP^Sc^ to mice, Aβ trimers promote brain deposition of soluble Aβ as amyloid plaques and accelerate the death of prion-infected mice, due to the onset of a mixed PrP^Sc^/Aβ pathology (Figure 3B) (Ezpeleta et al., 2019).

The inhibition of ROCK (or PDK1) in prion-infected neurons allows for the recovery of plasma membrane TACE α-cleavage activity toward PrP^C^ and APP, thus reducing the amyloid burden (PrP^Sc^, Aβ) and curing diseased neurons from neurotoxic proteins (Figure 4) (Pietri et al., 2013; Alleaume-Butaux et al., 2015; Ezpeleta et al., 2019). Such a reduction of the PrP^Sc^ level upon the inhibition of ROCKs would permit restarting of neuronal differentiation, beyond the relaxing effect of the inhibitors of ROCKs on the actin cytoskeleton (Relaño-Ginès et al., 2013). *In vivo*, the infusion of the ROCK inhibitor Y-27632 or the PDK1 inhibitor BX912 in prion-infected mice at late stages of the disease exerts beneficial effects in terms of improved motor activity (rod test) and increased survival time that correlate with restored protection against TNFα inflammation and reduced amounts of PrP^Sc^ and Aβ in the brain (Figure 4) (Pietri et al., 2013; Alleaume-Butaux et al., 2015; Ezpeleta et al., 2019).

## Prion Infection, Rock Oversignaling, and the Unfolded Protein Response

As mentioned above, ROCK overactivity caused by prion infection impacts the level of neurotransmitter-associated functions in 1C11^5−HT^ neuronal cells with a strong reduction of serotonin levels (Alleaume-Butaux et al., 2015). This is notably due to a lack of translation of the mRNA encoding Tryptophan Hydroxylase 2 (TPH-2), the key enzyme involved in the synthesis of serotonin. Because of the impact of ROCK overactivity on actin cytoskeleton dynamics, the deficit in TPH-2 expression would relate to the abnormal trafficking of TPH-2 mRNA in polarized neurons infected with prions and/or the incorrect organization of the translational machinery. Of note, ROCKs are known to activate by phosphorylation PTEN (for phosphate and tensin homolog) (Li et al., 2005), which in turn inhibits two critical positive regulators of protein synthesis, i.e., Akt and mTOR complex 1 (for the mammalian target of rapamycin) (Roffé et al., 2010; Simon et al., 2014). This pathway would additionally compromise the synthesis of TPH-2 in prion-infected neurons.

Only a few reports describe translational repression within a prion-infectious context induced by the Unfolded Protein Response (UPR) that occurs in the endoplasmic reticulum (ER) (Roffé et al., 2010; Moreno et al., 2013; Tanaka et al., 2020). The proteostress that exerts in the ER of prion-infected neurons provokes the activation by phosphorylation of the kinase PERK (PKR-like ER protein kinase) in the ER membrane, which in turn promotes the hyperphosphorylation of the translation factor eIF2α (eukaryotic initiation factor 2). Phosphorylated eIF2α then loses its active role in the translation of some mRNAs, which notably impairs the expression of pre-and post-synaptic proteins, provoking synapse failure and retraction, neuronal dysfunction and degeneration (Moreno et al., 2013).

**Figure 5:**
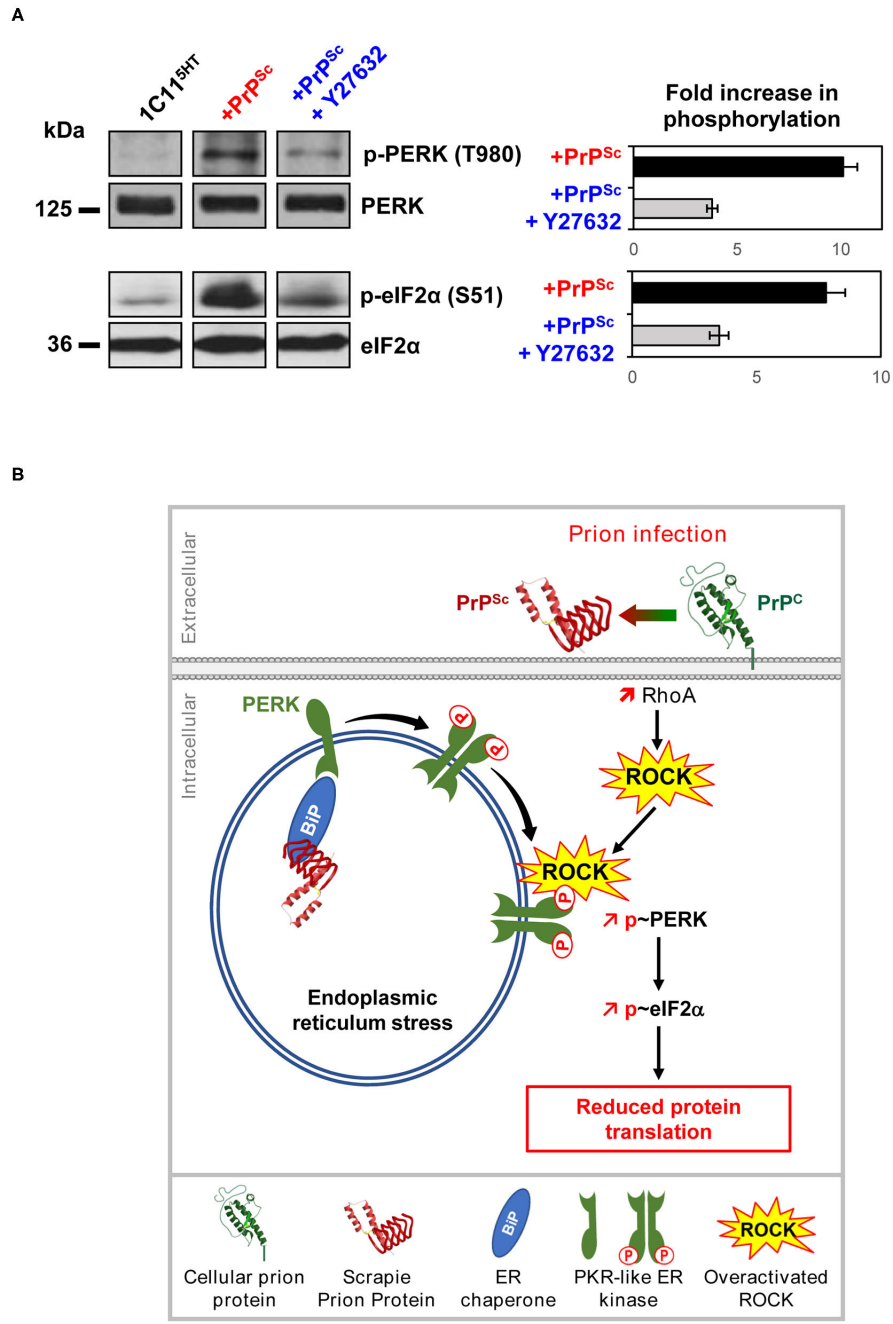
Deregulation of the PrP^C^-ROCK connection contributes to the endoplasmic reticulum stress and reduces protein translation in prion-infected neurons. **(A)** In 1C11^5−HT^ cells, PrP^Sc^ induces PERK and eIF2α phosphorylation. ROCK inhibition with Y-27632 (100 μM) reduces PrP^Sc^-induced phosphorylation of PERK and eIF2α, indicating that ROCKs contribute to the UPR activation pathway within a prion infectious context. **(B)** Model showing the implication of ROCKs in the Unfolded Protein Response in prion-infected neurons. In the endoplasmic reticulum (ER), PrP^Sc^ proteostress activates the chaperone BIP. Binding of BIP to the intraluminal tail of PERK then induces PERK dimerization and phosphorylation at the surface of ER. In parallel, overactivated ROCKs on prion infection attach to the ER membrane where ROCKs fuel and/or amplify PERK phosphorylation. Activated PERK then phosphorylates eIF2α leading to a reduction of protein translation.

We found that the rise of phosphorylation of PERK and downstream of eIF2α observed in prion-infected neurons is influenced by ROCK overactivity (Figure 5A). In serotonergic 1C11^5−HT^ neuronal cells infected by mouse-adapted human prions (Fukuoka strain), the inhibition of ROCKs with Y-27632 strongly reduces the PERK phosphorylation level, which restores, at least partly, eIF2α in its non-phosphorylated form active for translation (Figure 5A). This suggests that ROCKs are involved in the UPR response and take part in the control of the PERK-eIF2α pathway, which thus may explain at a mechanistic level how ROCKs govern the translation of some mRNAs. Such an impact of ROCK overactivity on the PERK-eIF2α pathway would account for rescued translation of TPH-2 mRNA and restoration of neuronal functions in prion-infected neurons exposed to the ROCK inhibitors (Figure 4). To our knowledge, these results introduce PERK, and thereby the translational initiation factor eIF2α, as novel effectors controlled by ROCKs. The ability of ROCKs to impact on the PERK pathway would relate to the property of ROCK, notably ROCK-2, to translocate from the cytosol to membranes following Rho-A-mediated activation (Figure 5B) (Leung et al., 1995). Whether PERK is a direct substrate of ROCK is unknown and how ROCK fits in within the UPR response requires further investigation.

## Corruption of the PrP^C^-Rock Connection in Other Amyloid-Based Neurodegenerative Diseases

Emerging evidence suggests that PrP^C^ could serve as a neurotoxic receptor in other unrelated neurodegenerative diseases, including Alzheimer's and Parkinson's disease. PrP^C^ was indeed shown to bind *in vitro* and *in vivo* Aβ and Tau of Alzheimer's disease (Laurén et al., 2009; Zou et al., 2011; Dohler et al., 2014; De Cecco et al., 2020), and pathological α-synuclein of Parkinson's disease (Aulić et al., 2017), and to relay, at least partly, the toxicity of those misfolded proteins and/or to promote their propagation in the diseased brain through connected neuronal structures. Because these overall pathological proteins display the common biochemical property to be enriched in β-sheet structures, it has been proposed that PrP^C^ would be a common sensor for all β-sheet rich protein conformers (Resenberger et al., 2011; Laurén, 2014). This may concern pathological proteins involved in other amyloid-based neurodegenerative diseases, such as superoxide dismutase 1 (SOD1), FUS or TDP-43 in Amyotrophic Lateral Sclerosis, or pathological Ataxin in Spinocerebellar Ataxia.

Concerning Alzheimer's disease, our work disclosed for the first time that Aβ peptides, through their interaction with PrP^C^, overactivate PDK1, which cancels TACE shedding activity toward TNFR1 and APP (Pietri et al., 2013). This not only renders Alzheimer's neurons highly vulnerable to TNFα inflammatory insult but also promotes the accumulation of Aβ40/42 peptides, before their deposition as senile plaques, in the brain of transgenic mice with an Alzheimer's like phenotype (Tg2576, 3xTgAD, 5xTgAD). The silencing of PDK1 in the brain of Alzheimer's mice, the infusion of the PDK1 inhibitor (Pietri et al., 2013), or the expression of a genetically inactivated PDK1 (Yang et al., 2017) exert protective effects by sustaining the neuroprotective cleavage activity of TACE toward TNFR1 and APP. From a behavioral point of view, antagonizing PDK1 activity improves memory and cognitive deficits in transgenic Alzheimer's mice (Pietri et al., 2013). As for prion infection, the rise in PDK1 activity in an Alzheimer's context originates from the perturbation of PrP^C^ signaling upon the binding of Aβ to PrP^C^. This likely promotes an excessive ROCK activity, and thereby an excess of ROCK-PDK1 complexes, at the root of TACE internalization and neutralization in Alzheimer's neurons. Although not explained at a mechanistic level in 2003, Zhou et al. already showed that inhibiting the Rho-ROCK pathway with Non-Steroidal anti-inflammatory drugs lowered the production of amyloidogenic Aβ42 in transgenic mice with Alzheimer's-like disease (Zhou et al., 2003). A decade after, Herskowitz et al. confirmed that the pharmacological inhibition of ROCK suppresses Aβ production in a mouse model with Alzheimer's disease (Herskowitz et al., 2013). SiRNA-based silencing of ROCK-2 was further shown to reduce Aβ42 cytotoxicity in primary cultures of cortical neurons (Liu et al., 2015) and to mitigate cognitive declines in a mouse model of Alzheimer's disease (Wen et al., 2014). Beyond a possible inhibitory effect of some ROCK-2 inhibitors on β-site APP cleaving enzyme 1 (BACE1) (Herskowitz et al., 2013), it is a good bet that the effect of those ROCK-targeting drugs on the level of Aβ42 relies on the dissociation of the ROCK-PDK1 complex and the subsequent rescue of the neuroprotective action of the α-secretase TACE on APP. As the PERK-eIF2α and LIMK/cofilin signaling pathways were also involved in Alzheimer's pathogenesis (Ma et al., 2013; Rush et al., 2018), we cannot exclude that the beneficial effect of drugs targeting ROCKs also depends on a rescue of protein synthesis, synapse maintenance (Henderson et al., 2019), and thereby preserved neurotransmission.

Beyond prion and Alzheimer's diseases, ROCKs were implicated in Parkinson's disease (Rodriguez-Perez et al., 2013; Labandeira-Garcia et al., 2015) and Amyotrophic Lateral Sclerosis (ALS) (Takata et al., 2013; Tönges et al., 2014). In mouse models with Parkinson's disease induced with either 1-Methyl-4-Phenyl-1,2,3,6-TetrahydroPyridine (MPTP) or 6-hydroxydopamine, the siRNA-based silencing or the genetic knockdown of ROCK-2, as well as the pharmacological inhibition of ROCKs with Y-27632, prolonged the survival of dopaminergic neurons in the *substantia nigra pars compacta* and significantly improved motor behavior (Tönges et al., 2012; Saal et al., 2015; Zhang et al., 2016). At a molecular level, ROCK inhibitors exert neuroprotective effects by rescuing neurite outgrowth and promoting regeneration (Tönges et al., 2012), stimulating dopamine metabolism (Tatenhorst et al., 2014), and/or up-regulating autophagy or mitophagy (Liu et al., 2016; Moskal et al., 2020). In a mouse model with an Amyotrophic Lateral Sclerosis-like disease, expressing G93A superoxide dismutase 1, the infusion of the ROCK inhibitor fasudil significantly delayed the onset of motor symptoms and increased the survival time of ALS mice, as a result of an increased number of surviving motor neurons in the spinal cord and a regenerative neuromuscular junction remodeling (Takata et al., 2013; Tönges et al., 2014). The beneficial effect of ROCK inhibition in ALS mice would mainly depend on rescued cytoskeletal functions. Based on the positive roles of ROCK inhibition toward ALS in preclinical models, the ROCK inhibitor fasudil entered a phase IIa clinical trial to probe the safety, tolerability, and efficacy of this drug in ALS patients at an early stage of disease (Lingor et al., 2019; Koch et al., 2020). According to the common biochemical and structural properties of pathological α-synuclein (Parkinson's disease) and SOD1 mutant (ALS), it is tempting to speculate that ROCK overactivity originates from the alteration of PrP^C^-mediated inactivation of small-G protein RhoA and ROCK on the binding of α-synuclein/SOD1 to PrP^C^.

## Conclusion

Although cellular prion protein PrP^C^ has been known for several decades for its pathological implication in prion diseases, the functions exerted by PrP^C^ remain an enigma. In this review, we document that PrP^C^, acting as a plasma membrane receptor or co-receptor, is an upstream regulator of the RhoA-ROCK signaling module. By toning down RhoA and ROCK activities, PrP^C^ orchestrates several cell functions, dealing with stem cell differentiation, the trafficking and translation of mRNAs, or the cell's protection against inflammation. From a molecular point of view, such a PrP^C^ depending on the control of ROCK activity is necessary for (i) the optimized dynamics of the actin cytoskeleton that accompanies the neuritogenesis process and contributes to the spatiotemporal activation of the translation machinery, (ii) the correct trafficking of cargo-vesicles and the distribution of neuroprotective factors and enzymes at the right cell place, and (iii) the movement of internal membranes involved in the clearance of wasting materials. The hijacking of the PrP^C^-RhoA-ROCK signaling pathway by pathogenic prions (PrP^Sc^) represents one of the first deleterious events in prion pathogenesis at the root of multiple adverse effects in neurons with the alteration of neuronal polarity, the hypervulnerability to inflammation, the activation of the unfolded protein response, the arrest of protein synthesis, and most importantly, the amplification of conversion of PrP^C^ into PrP^Sc^, creating a vicious cycle of conditions of PrP^Sc^ production by sustaining the activation of the RhoA-ROCK pathway. The phenotypic proximity between several unrelated amyloid-based neurodegenerative diseases, including Alzheimer's and Parkinson's diseases, and Amyotrophic Lateral Sclerosis, at the level of RhoA and ROCK overactivation, and the neuropathological consequences is intriguing. At least for Alzheimer's disease, PrP^C^ is the common denominator that overactivates the ROCK pathway on the binding of Aβ peptides to PrP^C^, leading to the accumulation and deposition of Aβ in the brain. Future studies are needed to address whether the hijacking of the PrP^C^-RhoA-ROCK pathway is a common, converging event of neurodegeneration in other amyloid-based neurodegenerative diseases.

The therapeutic options to combat those amyloid-based neurodegenerative diseases remain very limited and are even non-existing in the case of prion diseases. Whatever the neurodegenerative disease, the approved treatments are not curative, as they only delay the progression of the disease (symptom-modifying drugs). Prescription drugs for Alzheimer's disease include cholinesterase inhibitors (donepezil, rivastigmine, and galantamine), which impede the degradation of the acetylcholine neurotransmitter, and an N-methyl-D-aspartic (NMDA) acid receptor antagonist (memantine), for which the effectiveness is on par with the cholinesterase inhibitors. Memantine works for approximately half of individuals, but only for a short time (Zhang et al., 2020). Moreover, the side effects associated with the use of cholinesterase inhibitors includes vomiting, loss of appetite, nausea, and increased frequency of bowel movements. Headaches, constipation, confusion, and dizziness were reported in Alzheimer's patients treated with memantine. The leading drugs that delay Parkinson's disease are L-dopa (levodopa), whose metabolization into dopamine by the surviving dopaminergic neurons compensates for the lack of dopamine in the nigrostriatal axis, as well as inhibitors of the MonoAmine Oxidase type B (MAO-B)-dependent degradation of dopamine, such as rasagiline or amantadine (McFarthing et al., 2020). Parkinson's disease can also be treated with a multitude of other medications, including Catechol O-methyltransferase (COMT) inhibitors, anticholinergics, and dopamine agonists. They are, however, effective for a short time only, similar to Alzheimer's disease medications. Riluzole, a glutamatergic neurotransmission inhibitor, is the only drug approved for the treatment of ALS, but with modest benefits on patient survival (Jaiswal, 2019). An antioxidant drug, edaravone, was repurposed in ALS and approved in 2017 by the US Food and Drug Administration (Kiernan et al., 2021). Unfortunately, even if these two drugs are effective in slowing the progression of ALS, they cannot help fully cure the disease and are unable to revert ALS symptoms. On an experimental basis, other drugs (Masitinib, Ibudilast, Triumeq, Retigabine, and Tamoxifen) are currently being probed for their use in ALS treatments.

Amyloid-based neurodegenerative diseases are associated with multiple pathological processes, i.e., neurotransmission dysregulation, oxidative stress, autophagy, abnormal energetic metabolism, endoplasmic reticulum stress, and neuroinflammation; a combination of pathological events initiated by overproduced and accumulated amyloids. To develop disease-modifying drugs, preclinical and clinical studies are currently designed to reduce the amyloid burden (mainly based on active and passive immunotherapies) in the brain of diseased patients and/or to inhibit one or several pathological processes (Ballard et al., 2020; Cummings et al., 2020; Hung and Schwarzschild, 2020; McFarthing et al., 2020; Zhang et al., 2020). As the inhibition of ROCK decreases the production and accumulation of brain PrP^Sc^ and Aβ and blocks several steps in the amyloid pathocascades in prion and Alzheimer's diseases (Figure 4), ROCK inhibitors thus emerge as chemical candidates for curing amyloid-based neurodegenerative disorders.

## Author Contributions

All authors listed have made a substantial, direct and intellectual contribution to the work, and approved it for publication.

## Conflict of Interest

J-ML has non-financial competing interests with Hoffmann La Roche Ltd laboratories. He acts as an expert witness for Hoffmann La Roche Ltd laboratories. The remaining authors declare that the research was conducted in the absence of any commercial or financial relationships that could be construed as a potential conflict of interest.
